# An Epidemiological Study on the Prevalence of Atrial Fibrillation in the Chinese Population of Mainland China

**DOI:** 10.2188/jea.JE2008021

**Published:** 2008-10-01

**Authors:** Ziqiang Zhou, Dayi Hu

**Affiliations:** 1 Cardiovascular Center Beijing Tongren Hospital, Affiliate of Capital Medical University People’s Republic of China; 2 Heart Center People’s Hospital of Peking University People’s Republic of China

**Keywords:** Atrial Fibrillation, Prevalence, Epidemiological Study, Stroke, Logistic Regression

## Abstract

**Background:**

Atrial fibrillation (AF) is the most common arrhythmia encountered in clinical practice. Since only limited data on the Chinese population, which is the largest in the world, is available, we conducted an epidemiological study on the prevalence and risk factors of AF in mainland China.

**Methods:**

This population-based study conducted by cluster sampling comprised 29079 participants forming 14 cohorts from 13 provinces across China, where the population was nearly 1 billion. Every participant underwent electrocardiogram and physical examinations and responded to the interviewer-led questionnaire(s). Univariate and multiple statistical analyses were conducted to explore the relationship between AF prevalence and risk factors.

**Results:**

The age-standardized prevalence of AF in China (≥30 y) was 0.65%, and it increased with age. Men showed a higher prevalence of AF than women (0.91% [age-standardized, 0.66%] vs. 0.65% [0.63%], *P* = 0.013); several significant risk factors (age, hyperthyroidism, coronary heart disease, and rheumatic heart disease) were identified for AF in the general population. Stroke prevalence was much higher in AF patients than in non-AF people (12.95% vs. 2.28%, *P* < 0.001). AF was confirmed to be a significant independent risk factor for stroke prevalence in the studied population (OR = 2.776, [1.814, 4.248], *P* < 0.001). We found that AF patients received poor treatment (2.7%, warfarin; 39.7%, aspirin).

**Discussion:**

This study conducted on a large sample size demonstrates that AF prevalence in mainland China is slightly lower than that in Western countries and similar to that in Asian areas, and confirms that AF is a serious public health problem in China. We identified several potential risk factors, but their associations with AF still need to be further studied.

## INTRODUCTION

Atrial fibrillation (AF) is the most common sustained cardiac rhythm disturbance encountered in clinical practice. According to European and Northern American experiences, AF accounts for massive health care system expenses.^1^^,^^2^ The increased longevity is a result of improved medical care among patients with coronary artery disease, hypertension, and heart failure; these 3 chronic cardiac conditions generally predispose patients to AF. Several preliminary epidemiological studies based on US and European populations indicated that the prevalence of AF was on the increase with each passing decade.^3^^-^^5^

Epidemiological studies in Western countries have shown that in older people, the prevalence of AF increases with age from less than 1% for persons younger than 60 y of age to approximately 10% for those who are 80 or above.^3^^,^^6^^-^^8^ The Framingham Heart Study reported that men showed a higher AF prevalence than women.^5^ Data from the same study cohort also revealed AF to be an independent risk factor for stroke.^6^ Several risk factors of AF have been identified by studies based on white cohorts.^9^ These factors include age, congestive heart failure, valvular heart disease, male sex, a history of myocardial infarction, hypertension, and diabetes.^9^

For other ethnical populations, the Epidemiology, Practice, Outcomes, and Costs of Heart Failure (EPOCH) study indicated that the African-American race was associated with a lower prevalence of AF.^10^ Data from Asian countries like Japan,^11^^,^^12^ Singapore,^13^ and Korea^14^ also showed a lower prevalence of AF (0.7%-1.5%) compared to those from Western series. However, the prevalence of AF in China, which accounts for nearly one-fourth of the world population, has not been published in international journals. In order to clarify the prevalence of AF and explore its potential risk factors in China, we performed the first large-scale epidemiological study on AF in mainland China. This study provided data for analyzing the epidemiological characteristics of AF in the Chinese population, studying the association between stroke and AF, and comparing Chinese cohorts with other populations. The present study is an advanced version of the former preliminary study.^15^

## MATERIALS AND METHODS

### Study Population and Sample

A target sample size of approximately 30000 was calculated on the basis of the method introduced by Minassian DC,^16^ and the expected prevalence of AF (approximately 1%) was obtained from the preliminary data of the Western series.^3^^,^^6^^-^^8^

In the first stage, 14 centers based on 14 population-based cohorts from 13 provinces across China were established, such that they represented the average standards of living, education, and health care in mainland China. The cohorts were selected such that they represented the average characteristics of each province. There was no significant heterogeneity across these sites. The provinces studied included Guangdong, Hebei, Henan, Hubei, Hunan, Inner Mongolia, Shandong, Shanxi, Sichuan, Tianjin, Yunan, Zhejiang, and Jiangxi.

Given below is a map of the location of the clusters.

**Figure:**
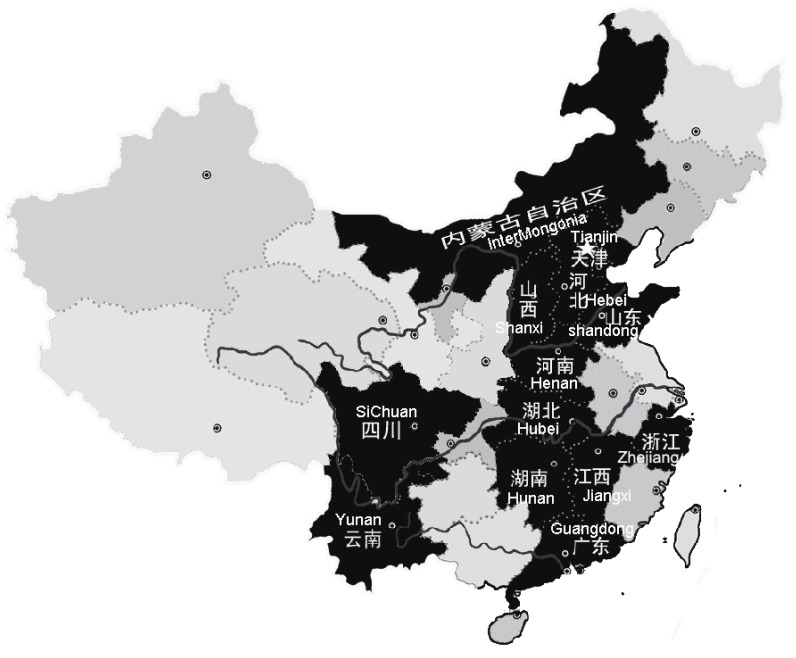


In the second stage, which was based on the data collected from the local household registration system in each chosen area, subjects were selected by cluster random sampling from each population established in the first stage. Each participating center was responsible for 2000-2500 subjects (born in or after 1972), and among the participants, a male to female ratio of 1:1 was required. The selected subjects were asked to come to the appointed center for examinations and interviews. This study was approved by the Health Ministry of the Peoples Republic of China and the local governments. A regular hospital or health agency in each area was appointed to provide the equipments and venues for this study. All the participants received a detailed introduction to this study by the local government and medical organizations. The participants’ informed consent was obtained by the local medical organizations. All the examinations were conducted free of charge, and no participant was compensated.

### Screening Process

We developed an interviewer-led questionnaire specifically for the survey. The first part of the questionnaire was recorded by the physicians, who conducted face-to-face interviews with the patients. The interviewers obtained information regarding the patients’ demographic characteristics and recorded the family medical history; they inquired about their smoking habits; drinking habits; and a medical history of other diseases, including diabetes, hypertension, dyslipidemia, rheumatic heart disease (RHD), coronary heart disease (CHD), hyperthyroidism, stroke, and AF. Besides, participants with self-reported RHD underwent further physical examinations and echocardiography if the diagnosis was unclear. We did not routinely perform blood tests for thyroid function, blood sugar, and blood lipid. Participants with a self-reported history of stroke were all required to provide their medical records from hospital visits and/or visits to primary care practitioners, which were carefully reviewed by qualified local neurologists. The second part of the questionnaire was recorded by 2 trained technicians who obtained the blood pressure, pulse rate, height, weight, waist, and hip measurements. After asking the patient to rest for at least 10 min in the sitting position, the blood pressure in the right arm was measured in the sitting position with the use of a standardized mercury column sphygmomanometer with a standardized protocol.^17^ The third part included a copy of an electrocardiogram (ECG) with a written interpretation. Patients with AF and suspected AF were required to provide information on the use of anticoagulants.

Hypertension was defined by a systolic blood pressure of 140mmHg or higher, diastolic blood pressure of 90mmHg or higher, the use of antihypertensive agents, or a combination of these. Participants were considered to be smokers if they were smokers at the time of the investigation or if they had quit smoking less than 6 month earlier. They were considered non-drinkers if they did not consume alcohol regularly or had quit for not less than 6 months. Diagnoses of diabetes, hyperthyroidism, dyslipidemia, and CHD were classified by physicians (interviewers) as “yes” on the basis of positive self-reported histories of these diseases. Education attainment was classified as “yes” if a subject was a high school graduate or had higher education.

Several steps were used to ensure standardization of the methods: (1) Physicians and technicians from each center received unified training prior to the screening process. (2) All participants were asked to respond to the interviewer-led questionnaire. (3) Blood pressure, pulse rate, height, weight, waist, and hip measurements were recorded by trained technicians. (4) All participants underwent an ECG examination, which was performed by trained technicians and trained physicians in each center. The physicians provided a primary written interpretation for each ECG, all of which were reviewed by an appointed senior cardiologist daily. (5) Monitors were sent to each center to supervise the standardized procedures. (6) Trained interviewers contacted those who did not come to the interview by telephone, and then conducted a family interview wherever possible to encourage response from the patient. The screening work for each center was performed within 3 months in 2003.

### Definition of AF

Subjects who were diagnosed with AF during the ECG examination in the screening process were defined as AF cases. Those who were not found to have AF according to the ECG test, but had previous medical records from visits to qualified affiliated hospital(s) of medical universities or any prior ECG record(s) for AF episode(s) were also defined as having AF (possible paroxysmal AF^18^^,^^19^). AF determination based on the ECG was first conducted by a physician, who acted as the interviewer, and then verified by an appointed senior physician or cardiologist. ECGs and medical records obtained outside the study center were copied for further verification. The ECG criteria and classification for AF were in accordance with the ACC/AHA/ESC 2001 guidelines.^19^

In order to ensure that the maximum number of AF cases were identified, suspected patients for whom AF was not detected according to the ECG but who were able to recall AF or tachycardia episode(s), which might have been diagnosed by primary care practitioners or ECG technicians, received further reviews of their medical records within 1 y from hospital visits, visits to primary care practitioners, and other potential evidences. Their health records, if present, were also fully searched in the database of local medical organizations. Then, all these suspected AF cases were further investigated by senior cardiologists to confirm or disconfirm the diagnosis.

### Statistical Analysis

We used Borland Delphi 6.0 to build a database management system to handle the data obtained in this study. Data analysis was conducted with the SPSS statistical software, version 12.0.1. Differences between groups were tested with Pearson’s χ^2^ test or Fisher’s exact test when appropriate. The exact confidence intervals (C.I.) of the prevalence were calculated by StatPages.net (http://statpages.org/confint.html) based on binomial distribution. We calculated the overall as well as age- and sex-stratified point prevalence of AF in the participants.^20^ The direct age standardization method^20^ was chosen to calculate the age-standardized prevalence and expected number of AF patients in the 13 studied provinces and in all of China, on the basis of the standard age distribution data from China’s fifth national population census in 2000.

Two main multiple logistic regression models were constructed. In one model, the dependent variable was AF (yes/no), and the backward stepwise method was used to investigate the associations between the potential risk factors and AF prevalence. For potential independents, we defined age and waist-hip ratio (WHR) as continuous variables and the following as binomial categorical independent variables: sex (male/female), diabetes (yes/no), hyperthyroidism (yes/ no), hypertension (yes/no), RHD (yes/no), CHD (yes/no), smoking (yes/no), drinking (yes/no), dyslipidemia (yes/no), and education attainments (high school graduate or not). Univariate analyses were first conducted to test the correlation between AF prevalence and each potential independent variable. In the univariate analysis, variables with *P* < 0.25 were chosen as independent variables to construct the model for multivariable analyses (backward stepwise logistic regression model). The other logistic model was used to explore the association between AF and the prevalence of stroke, where the dependent variable was stroke (yes/no) and the independent variables based on previous studies^21^ included age, diabetes, hypertension, dyslipidemia, smoking, and drinking. In the backward stepwise logistic regression model, the criterion for inclusion of the variables was *P* < 0.05, and that for exclusion was *P* > 0.10. The values for odds ratios (ORs) were linked to the prevalence of AF, i.e., values greater than 1 indicated an increased risk of high AF prevalence and values less than 1 indicated a reduced risk of high AF prevalence.

## RESULTS

The study included 29079 subjects (mean age, 52.5 ± 22.4; range, 30-100) from 14 different Chinese cohorts (male, n = 13558; female, n = 15521), among which 224 participants were identified as having AF. The overall response rate was approximately 96%. The most common reason for nonparticipation (4%) was refusal to undergo examination. Among the 224 AF patients, 204 (91.1%) were identified on the basis of the ECGs during the screening process, and the rest (20, 8.9%) were identified via previous medical records and ECGs. Prior to data analysis, we ensured that all participants were examined for AF or suspected AF. Our results showed that the crude prevalence of AF in the studied Chinese population (≥30 y) was 0.77% (95% C.I., 0.67-0.88%). On the basis of the age composition data in the 13 studied provinces and all of China (obtained from the fifth national population census in China), the age-standardized prevalence of AF in both genders in the Chinese (≥30 y) was 0.65% (0.66% for males and 0.63% for females); the estimated number of AF patients (≥30 y) was calculated to be 2456573 in all the provinces studied and 4193857 in all of China.

The age-specific prevalence of AF increased with age (see Table 1). The crude prevalence of AF in men was higher than that in women (0.91% vs. 0.65%, *P* = 0.013). In each age group, the prevalence of AF in men was higher than that in women, except for the 50-59 age group (see Table 1).

**Table 1.** Sex- and age-specific (by 5 y) prevalence of AF in the Chinese

Age	Male					Female					Sum					Population in 13 Provinces*		

	AF^†^ (n)	N	%	95% C.I.		n	N	%	95% C.I.		n	N	%	95% C.I.		Male	Female	Overall
		
				Lower	Upper				Lower	Upper				Lower	Upper			
30-34	0	86	0	-	-	0	164	-	-	-	0	250	0	-	-	38489327	36474295	74963622
35-39	0	1818	0	-	-	0	2476	-	-	-	0	4294	0	-	-	32724316	30997573	63721889
40-44	4	1633	0.24	0.07	0.63	1	1913	0.05	0.00	0.29	5	3546	0.14	0.05	0.33	24590274	22677583	47267857
45-49	6	2051	0.29	0.11	0.64	7	2482	0.28	0.11	0.58	13	4533	0.29	0.15	0.49	25689746	24283278	49973024
50-54	11	2063	0.53	0.27	0.95	14	2539	0.55	0.30	0.92	25	4602	0.54	0.35	0.80	19294500	17825684	37120184
55-59	8	1756	0.46	0.20	0.90	10	1808	0.55	0.27	1.01	18	3564	0.51	0.30	0.80	13842508	12691301	26533809
60-64	16	1483	1.08	0.62	1.75	17	1701	1.00	0.58	1.60	33	3184	1.04	0.71	1.45	12513522	11418134	23931656
65-69	25	1360	1.84	1.19	2.70	17	1336	1.27	0.74	2.03	42	2696	1.56	1.12	2.10	10261096	10073564	20334660
70-74	24	796	3.02	1.94	4.45	17	659	2.58	1.51	4.10	41	1455	2.82	2.03	3.80	7282788	7776278	15059066
75-79	17	353	4.82	2.83	7.60	8	308	2.60	1.13	5.05	25	661	3.78	2.46	5.53	4204410	5236874	9441284
80-84	6	120	5.00	1.86	10.57	7	100	7.00	2.80	14.40	13	220	5.91	3.18	9.89	1859090	2859173	4718263
≥85	6	39	15.38	5.86	30.53	3	35	8.57	1.80	23.6	9	74	12.16	5.71	21.84	760967	1589690	2350657

**Total**	**123**	**13558**	**0.91**	**0.75**	**1.08**	**101**	**15521**	**0.65**	**0.53**	**0.79**	**224**	**29079**	**0.77**	**0.67**	**0.88**	**191512544**	**183903427**	**375415971**

*This included the overall population in the 13 provinces studied, where the corresponding age strata of the general Chinese population were cited from the fifth national population census conducted in November 2000 in China; ^†^: atrial fibrillation

In the 224 participants with AF, only 6 took warfarin (see Table 2), and only 1 of these 6 underwent an internalized normal ratio examination regularly.

**Table 2.** Information on the use of medicine by Chinese AF* patients

Medicine	None		Rarely (<3 times per week)		Frequently (≥3 times per week)		Sum (rarely+frequently)	

	n	%	n	%	n	%	N	%
Warfarin	218	97.3	2	0.9	4	1.8	6	2.7

Aspirin	139	62.1	40	17.9	45	20.1	85	37.9
Digoxin	139	62.1	59	26.3	26	11.6	85	37.9
*β*-blockers	169	75.4	37	16.5	18	8.0	55	24.6

*: atrial fibrillation

The baseline characteristics of the variables and the results from the univariate analyses are listed in Table 3. Drinking and smoking were then excluded due to a very high *P* value. The remaining 9 variables were independent variables that were entered into the logistic regression model in the first step.

**Table 3.** Baseline characteristics of the variables included in the multivariable model

	AF* (n = 224)	%	95% C.I.	Non-AF (n = 28855)	%	95% C.I.	*P*
Smoking	88	39.29	32.85-42.01	10303	35.71	35.15-36.26	0.294
Drinking	82	36.61	30.29-43.28	10244	35.50	34.95-36.06	0.782
Diabetes	13	5.80	3.13-9.72	1009	3.50	3.29-3.72	0.065
Hyperthyroidism	6	2.68	0.99-5.74	357	1.24	1.11-1.37	0.103
Dyslipidemia	42	18.75	13.86-24.49	3281	11.37	11.01-11.74	0.001
Hypertension	121	54.02	47.25-60.68	10314	35.74	35.19-36.30	<0.001
CHD^†^	29	12.95	8.84-18.06	1085	3.76	3.54-3.99	<0.001
RHD^‡^	29	12.95	8.84-18.06	62	0.21	0.16-0.28	<0.001
Age ≥ 60	163	72.77	66.44-78.48	7967	27.61	27.10-28.13	<0.001
Age (mean±*SD*)^§^	65.74 ± 10.98	-	-	52.43 ± 11.40	-	-	<0.001
Sex (male)	123	54.91	48.14-61.55	13435	46.56	45.98-47.14	0.013
Education attainment	52	23.21	17.85-29.30	9860	34.17	33.62-34.72	<0.001

*: atrial fibrillation; ^†^: coronary heart disease; ^‡^: rheumatic heart disease; ^§^: mean ± standard deviation

Four significant risk factors, including age, hyperthyroidism, RHD, and CHD, were located via the backward stepwise logistic regression model (see Table 4). Among the variables that were entered in the last step of the regression, neither dyslipidemia nor sex was found to be statistically significant on the basis of the criteria (*P* < 0.05) (see Table 4). WHR (per 0.1) and waist circumference (per cm) were significantly associated with the prevalence of AF when it was not adjusted for other factors (OR = 1.173, [1.041, 1.322], *P* = 0.009; OR = 1.020, [1.008, 1.033], *P* = 0.002), but they were statistically insignificant after adjustment. As another anthropometric index, body mass index (BMI) was not significantly associated with AF prevalence even when it was not adjusted with the other variables (*P* = 0.552). WHR (per 0.1) became a significant risk factor for AF (OR = 1.212, [1.010, 1.456], *P* = 0.039) when only the male population was considered. Collinearity diagnosis was routinely performed, and no independent variable was found to have a tolerance of <0.1. Analysis of variance inflation factor (VIF), Eigenvalue, and the condition index did not show significant multicollinearity.

**Table 4.** Risk factors of AF in both genders in the Chinese population in mainland China

	*P*	OR	95% C.I. for OR	

			Lower	Upper
Age (per year)	<0.001	1.104	1.090	1.119
Hyperthyroidism (yes)	0.014	2.861	1.240	6.607
CHD (yes)	<0.001	2.245	1.486	3.395
RHD (yes)	<0.001	97.116	58.022	162.712
Sex (male)	0.052	1.317	0.997	1.737
Dyslipidemia (yes)	0.062	1.316	0.983	1.996

During the backward stepwise regression, the variable(s) removed from steps 2, 3, 4, and 5 were hypertension, diabetes, education attainment, and WHR respectively.

In the univariate analysis, the prevalence of stroke in AF patients was much higher than that in the non-AF population (12.95% vs. 2.28%, *P* < 0.001) (See Table 5a). Moreover, in the logistic regression model, AF was proven to be one of the leading contributors to the prevalence of stroke (OR = 2.776, [1.814, 4.248], *P* < 0.001) (See Table 5b).

**Table 5a.** Prevalence of stroke in AF and non-AF cohorts

	Stroke (n)	Non-Stroke (n)	Total subjects (N)	Prevalence of stroke (%)	95% C.I.
AF	29	195	224	12.95	8.84-18.06
Non-AF	657	28173	28855	2.28	2.11-2.46
Sum	686	28368	29079	2.36	2.19-2.54

**Table 5b.** AF* as an independent variable of the prevalence of stroke in the studied Chinese population

	*P*	OR^†^	95.0% C.I. for OR	

			Lower	Upper
Dyslipidemia (yes)	<0.001	3.220	2.717	3.815
Hypertension (yes)	<0.001	3.067	2.543	3.698

AF (yes)	<0.001	2.776	1.814	4.248
Age (per year)	<0.001	1.063	1.055	1.071
Diabetes (yes)	<0.001	1.924	1.505	2.459
Smoking (yes)	<0.001	1.460	1.211	1.761
Drinking (yes)	0.510	0.937	0.773	1.137

*: atrial fibrillation; ^†^: odds ratio

## DISCUSSION

This study demonstrates that the age-standardized prevalence of AF in the general Chinese population was 6.5 per 1000 people, and that it increased with age. Given China’s 6.4 billion population (≥30 y), it has been estimated that at least 4 million adults suffer from AF in mainland China today, which can be a substantial burden on public health. Here, we confirm that AF is a disease that is largely prevalent in the elderly in the general Chinese population, with the great majority of cases present among people aged 60 y and above.

The age-specific prevalence of AF in China rose dramatically from 0% in the 30-39 age group to 7.5% in the 80-89 age group. Similar trends were found in nearly all the previous studies on AF prevalence. The present study showed a lower prevalence rate of AF in Chinese people, especially in the middle-aged cohort, than in Western cohorts.^3^^,^^5^^-^^8^^,^^22^^-^^24^ Firstly, it should be brought to notice that ascertaining the diagnosis of AF and the characteristics of the population studied would significantly affect the final result of a study. Wolf et al.^5^ included the AF cases diagnosed from ECGs that were obtained from the subjects’ private physicians and during interim hospitalizations. Inclusion of such AF cases could prevent the loss of cases with acute paroxysmal AF, which may have occurred^25^ in the present or Friberg’s study.^22^ Majeed et al.^3^ used a large and well-validated general practice-derived database to diagnose AF, which is more sensitive than the single ECG used in the present study; moreover, they gained baseline data from clinical practices when there was a lack of completely healthy subjects, and therefore, they tended to obtain a higher prevalence of AF. AF prevalence according to a community study on middle-aged to elderly Japanese people was only 1.3%,^12^ whereas that in ill, hospitalized, elderly people could reach as high as 22%.^26^ As such, the lower prevalence of related diseases, such as coronary artery disease, diabetes, and dyslipidemia, in the population studied contributed to a lower AF prevalence. Secondly, the use of different data sets is also an important reason for the difference. It has recently been indicated that AF prevalence varies according to race,^27^ which may partly explain why AF prevalence found in this study is much closer to the results from Asian cohorts^10^^-^^14^ outside China than to those from the Western series. Moreover, another essential reason is the different age strata among the studies, because AF increases with age. Studies based on younger populations often indicated a lower prevalence of AF,^1^^,^^12^^,^^28^^,^^29^ and those based on older participants showed a higher prevalence.^6^^,^^23^^,^^24^^,^^30^^,^^31^ In conclusion, it can be said that AF is a disease that is increasing in prevalence;^5^ therefore, it should be noted that the prevalence of AF obtained in this study may actually be lower than that reported in previous studies.

We found no significant association between smoking or drinking and the prevalence of AF. Kannel WB et al. reported that smoking was a significant risk factor in women when adjusted only for age.^32^ Luc Djousse et al. concluded that long-term alcohol consumption was a strong predictor of the incidence of AF adjusted for other risk factors.^7^ The reason as to why no significant association was found between AF prevalence and these lifestyle-related factors was probably due to the cross-sectional design of the present study.

In this study, hypertension was not significantly associated with the prevalence of AF, whether it was adjusted only for age or for all risk factors, although it was by far a powerful predisposing factor for AF incidence.^7^^,^^29^^,^^30^ Hypertension is a major contributing factor to the development of AF in longitudinal studies, but it is not always a risk factor for AF in cross-sectional analyses. Patients who have developed AF usually tend to show a lowering of systolic blood pressure (“decapitated” hypertension) compared to the blood pressure levels prior to the onset of AF. Second, some drugs used for the treatment of hypertension also suppress AF, such as beta-blocker and certain calcium channel blockers. These factors may attenuate the relationship between elevated blood pressure and the prevalence of AF in a cross-sectional analysis.

Bailey et al. indicated that estrogen plays a role in the occurrence of AF.^33^ Considering that the proportion of menopause almost reaches 100% among women aged 60 y or above and increases with age among women aged 30-59 y (see Table 1), we analyzed the female subgroup aged 35-62 y (this group included the youngest as well as the oldest menopausal females) but found no significant association between AF and menopause (*P* = 0.59). Educational attainment was not significantly associated with AF prevalence in this study. The older Chinese people are, the lower their average level of educational attainment; this may affect the result.

On the basis of the present study, we found a significantly higher prevalence of stroke in subjects with AF than in non-AF subjects. In the multiple logistic regression model, AF was also confirmed to be one of the leading contributors to the prevalence of stroke after adjustment for age and several other known risk factors. Therefore, the strong link between AF and stroke was confirmed in the Chinese population studied. Due to the much higher prevalence of stroke in AF cases, anticoagulation therapy is highly recommended in China for stroke prevention in patients with AF. Unfortunately, the number of AF patients who took anticoagulants regularly (2.7%, warfarin and 37.9%, aspirin) was rather small. Health education and public policies are urgently required for Chinese AF patients.

The present study had some limitations. Firstly, we did not find a significant association between AF and several predisposing factors. The reasons may be due to both the cross-sectional study and the relatively low number of diagnosed AF participants. Secondly, the prevalence of diabetes was very low (3.5%, [3.3%-3.7%]) compared with that of hypertension (35.9%, [35.3%-36.4%]), which may reflect the lack of biochemical examinations. Only 91 (0.3%) of 29079 participants were found to have RHD, which may explain the wide confidence intervals for RHD. Moreover, although we obtained a single ECG recording for each participant and tried to obtain complete information from those diagnosed with AF, it was still hard to discover paroxysmal AF in some cases; this limitation is also true for other studies. Therefore, the prevalence of AF revealed in this study may represent the lower boundary of the true prevalence. With regard to the Chinese, though Dr. Qi et al. and Dr. Yap et al. reported some results based on hospitalized patients^34^ and community-dwelling populations,^13^ respectively, no data on the general population has demonstrated the incidence of AF and the correlation between it and the risk factors; this area requires to be explored by a carefully designed follow-up study.
